# Insulin promotes invasion and migration of *KRAS^G12D^* mutant HPNE cells by upregulating MMP‐2 gelatinolytic activity via ERK‐ and PI3K‐dependent signalling

**DOI:** 10.1111/cpr.12575

**Published:** 2019-03-05

**Authors:** Guangfu Wang, Lingdi Yin, Yunpeng Peng, Yong Gao, Hao Gao, Jingjing Zhang, Nan Lv, Yi Miao, Zipeng Lu

**Affiliations:** ^1^ Pancreas Center First Affiliated Hospital of Nanjing Medical University Nanjing China; ^2^ Pancreas Institute, Nanjing Medical University Nanjing China

**Keywords:** gelatinolytic activity, insulin, migration and invasion, MMP‐2

## Abstract

**Objectives:**

Hyperinsulinemia is a risk factor for pancreatic cancer, but the function of insulin in carcinogenesis is unclear, so this study aimed to elucidate the carcinogenic effects of insulin and the synergistic effect with the *KRAS* mutation in the early stage of pancreatic cancer.

**Materials and methods:**

A pair of immortalized human pancreatic duct‐derived cells, hTERT‐HPNE E6/E7/st (HPNE) and its oncogenic *KRAS^G12D^* variant, hTERT‐HPNE E6/E7/*KRAS^G12D^*/st (HPNE‐mut‐*KRAS*), were used to investigate the effect of insulin. Cell proliferation, migration and invasion were assessed using Cell Counting Kit‐8 and transwell assays, respectively. The expression of E‐cadherin, N‐cadherin, vimentin and matrix metalloproteinases (MMP‐2, MMP‐7 and MMP‐9) was evaluated by Western blotting and/or qRT‐PCR. The gelatinase activity of MMP‐2 and MMP‐9 in conditioned media was detected using gelatin zymography. The phosphorylation status of AKT, GSK3β, p38, JNK and ERK1/2 MAPK was determined by Western blotting.

**Results:**

The migration and invasion ability of HPNE cells was increased after the introduction of the mutated *KRAS* gene, together with an increased expression of MMP‐2. These effects were further enhanced by the simultaneous administration of insulin. The use of MMP‐2 siRNA confirmed that MMP‐2 was involved in the regulation of cell invasion. Furthermore, there was a concentration‐ and time‐dependent increase in gelatinase activity after insulin treatment, which could be reversed by an insulin receptor tyrosine kinase inhibitor (HNMPA‐(AM)_3_). In addition, insulin markedly enhanced the phosphorylation of PI3K/AKT, p38, JNK and ERK1/2 MAPK pathways, with wortmannin or LY294002 (a PI3K‐specific inhibitor) and PD98059 (a MEK1‐specific inhibitor) significantly inhibiting the insulin‐induced increase in MMP‐2 gelatinolytic activity.

**Conclusions:**

Taken together, these results suggest that insulin induced migration and invasion in HPNE and HPNE‐mut‐*KRAS* through PI3K/AKT and ERK1/2 activation, with MMP‐2 gelatinolytic activity playing a vital role in this process. These findings may provide a new therapeutic target for preventing carcinogenesis and the evolution of pancreatic cancer with a background of hyperinsulinemia.

## 1 INTRODUCTION

Pancreatic ductal adenocarcinoma (PDAC) is a lethal digestive malignancy, and its overall 5‐year survival is less than 8%. It is the fourth most common cause of cancer‐related death in the United States.^1^ Although the incidence of pancreatic cancer has increased recently, the survival rate has not improved significantly.^2^ Surgical resection is the only curative treatment for pancreatic cancer, but the surgical excision rate is less than 20% due to poor early diagnosis.^3^ Therefore, a better understanding of the molecular mechanisms governing pancreatic cancer carcinogenesis is required for the prevention, early diagnosis and treatment of pancreatic cancer.

The mutation of the *KRAS* proto‐oncogene is thought to be an initiating genetic lesion in the stepwise progression of pancreatic cancer.^4^ Previous studies revealed that the increasing *KRAS* mutation frequency correlated with the PanIN stage and it is nearly universal (>95%) in human PDAC.^5^, ^6^ Moreover, transgenic mouse models confirmed that the *KRAS^G12D^* mutation can reprogramme cells into a duct‐like fate, which, in turn, induces acinar‐to‐ductal metaplasia, pancreatic intraepithelial neoplasia (PanINs) and, ultimately, PDAC.^6^, ^7^ Interestingly, in another mouse model with a *KRAS^G12V^* mutation, PanINs could be only induced if chronic inflammation and mutation existed at the same time.^8^ These studies suggested that the occurrence of pancreatic cancer is more likely to be a combination of genetic and non‐genetic events.

Growing evidence indicates that there is a close connection between type 2 diabetes and the increased incidence of pancreatic cancer.^9^, ^10^ It has been reported that half of the patients with pancreatic cancer have diabetes and a large sample cohort study suggested a 2.17‐fold risk of pancreatic malignancy in type 2 diabetic patients.^12^, ^13^

In addition, studies in genetically engineered mouse models have also shown that oncogenic *KRAS *can induce mPanIN spontaneously^14^ and that type 2 diabetes caused by a high‐fat, high‐calorie diet can accelerate the development of precancerous lesions.^15^ Numerous studies have investigated how insulin, rather than blood glucose, is an independent risk factor for pancreatic cancer.^16^, ^17^ However, the direct contribution of hyperinsulinemia to the increased incidence of pancreatic cancer in type 2 diabetes remains unclear. In this study, we explored the role of insulin in the malignant progression of human pancreatic duct‐derived cells and the underlying mechanism.

## 2 MATERIALS AND METHODS

### 2.1 Chemicals and reagents

Insulin and pork gelatin were obtained from Sigma‐Aldrich (St. Louis, MO). Antibodies to the insulin receptor β (IRβ) subunit (C‐terminus) and hydroxy‐2‐naphthalenylmethylphosphonic acid trisacetoxymethyl (HNMPA‐(AM)_3_), an insulin receptor tyrosine kinase inhibitor, were obtained from Abcam (Cambridge, MA). The remaining antibodies were purchased from Cell Signaling Technology (Danvers, MA). SB203580 (a p38 inhibitor), wortmannin (a PI3K inhibitor), LY294002 (a PI3K inhibitor) and its control LY303511, rapamycin (an mTOR/p70^s6^ kinase inhibitor), PD98059 (a MEK1 inhibitor), SP600125 (a JNK inhibitor) and picropodophyllin (PPP, an IGF1R inhibitor) were obtained from Selleck Chemicals (Houston, TX).

### 2.2 Cell lines and cell culture

The telomerase‐immortalized hTERT‐HPNE (Human Pancreatic Nestin‐Expressing ductal cells) E6/E7/st (HPNE) and its oncogenic *KRAS* variant, hTERT‐HPNE E6/E7/*KRAS^G12D^*/st (HPNE‐mut‐*KRAS*), were obtained from the American Type Culture Collection (ATCC). The hTERT‐HPNE cells can be transformed through the stepwise introduction of oncogenes designed to mimic PDAC progression, including oncogenic *KRAS* (carrying the G12D mutation), HPV16 E6 and E7 proteins (to abrogate p53 and RB), and the SV40 small‐t antigen (to inhibit PP2A). Cell lines were cultured in the recommended complete growth medium, which included 5% foetal bovine serum, 75% DMEM without glucose (Sigma Cat #D‐5030), 25% Medium M3 Base (Incell Corp. Cat #M300F‐500), 10 ng/mL of human recombinant EGF, 5.5 mmol/L of D‐glucose (1 g/L) and 750 ng/mL of puromycin in the presence of 5% CO_2_ at 37°C.

### 2.3 RNA isolation and quantitative real‐time PCR

Total RNA was isolated from cells using TRIzol reagent (Life Technologies, Carlsbad, CA) according to the manufacturer's protocol. Then, the RNA was reverse‐transcribed using PrimeScript RT Master Mix (Takara, Tokyo, Japan). RT‐qPCR was performed to detect the mRNA expression with FastStart Universal SYBR Green Master (Roche, IN), using β‐actin as the loading control. The MMP‐2 (matrix metalloproteinases 2) and β‐actin primers were as follows: for MMP‐2, 5′‐TAC AGG ATC ATT GGC TAC ACA CC‐3′ (sense) and 5′‐GGT CAC ATC GCT CCA GAC T‐3′ (antisense); and for β‐actin, 5′‐AGC GAG CAT CCC CCA AAG TT‐3′ (sense) and 5′‐GGG CAC GAA GGC TCA TCA TT‐3′ (antisense).

### 2.4 Transfection of small interfering RNA

The siRNAs used in the study were synthesized by GenePharma (Shanghai, China), and their sequences were as follows: for MMP‐2 siRNA#1, 5′‐GUG GCC AAC UAC AAC UUC UTT3′ (sense) and 5′‐AGA AGU UGU AGU UGG CCA CTT‐3′ (antisense); for MMP‐2 siRNA#2, 5′‐GCA CCC AUU UAC ACC UAC ATT‐3′ (sense) and 5′‐UGU AGG UGU AAA UGG GUG CTT‐3′ (antisense); for MMP‐2 siRNA#3, 5′‐GCA GAC AUC AUG AUC AAC UTT‐3′ (sense) and 5′‐AGU UGA UCA UGA UGU CUG CTT‐3′ (antisense); and for the negative control, 5′‐UUC UCC GAA CGU GUC ACG UTT‐3′ (sense) and 5′‐ACG UGA CAC GUU CGG AGA ATT‐3′ (antisense). The jetPRIME^®^ transfection reagent (Polyplus, ILLKIRCH, France) was applied to transfect both cell lines (final conc. of 20 nmol/L). The efficiency of all siRNAs was validated by qRT‐PCR and gelatin zymography.

### 2.5 Cell proliferation assay

The HPNE and HPNE‐mut‐*KRAS* cells were seeded into 96‐well plates at a density of 1.5 × 10^3^ cells per well. The premixed medium (10 μL of Cell Counting Kit‐8 reagent (Dojindo, Tokyo, Japan), 100 μL of medium) was added to each well. After incubation at 37°C for 3 hours in the dark, the absorbance of each well was measured at 450 nm to detect the cell viability via a microplate reader.

### 2.6 Migration and invasion assays

The impact of insulin on cell migration and invasion was assessed using transwell filters (8.0 μm) purchased from BD Biosciences (Franklin Lakes, NJ). HPNE (2 × 10^4^) and HPNE‐mut‐*KRAS* (2 × 10^4^) cells were seeded into the upper chamber containing an uncoated or Matrigel‐coated membrane. Serum‐free medium (200 μL) was added to the upper chamber, and complete medium (500 μL) was added to the lower chamber. Cells that migrated to the lower compartment and adhering to the bottom surface of the membrane were stained with crystal violet after incubation at 37°C in a humidified 5% CO_2_ incubator, and the non‐migrating cells are removed from the upper surface of the membrane by “scrubbing.” Three high‐power (200×) fields per well were randomly photographed, and the number of cells adhering to the bottom surface of the membrane was counted. The number of migrated HPNE cells without insulin treatment on the bottom surface of the membrane was used as a basal control.

### 2.7 Western blot analysis

Briefly, protein was extracted using a total protein extraction kit (Keygen BioTECH, Nanjing, China). The mixed ice‐cold lysis buffer contains the following reagents: 1 mL lysis buffer, 10 μL 100 mmol/L PMSF, 1 μL protease inhibitors and 10 μL phosphatase inhibitors. The extracted protein was mixed with 5× SDS and boiled. Standard methods were utilized to analyse protein expression,^18^ and β‐actin was used as a loading control.

### 2.8 Gelatin zymography

Both cell lines were grown to 80% confluence and then incubated in serum‐free medium. All inhibitors as indicated in the figure legends were added 2 hours prior to insulin, and the cells were allowed to grow for 24 hours. The conditioned medium was concentrated using the Centricon‐10 system. Quantified amounts (20 mg in each well) of conditioned medium were loaded in a 10% SDS gel containing 1 mg/mL of gelatin. β‐Actin was used as a loading control. The gel was washed for 1 h in a solution of Triton X‐100 (2.5%) in order to exchange SDS and renature the enzyme. The gel was then incubated for 24 hours at 37°C in a buffer (0.05 mol/L of Tris‐HCl, pH 7.5; 5 mmol/L of CaCl_2_; 1 μmol/L of ZnCl_2_; 0.02% Brij‐35) so as to promote MMPs to degrade gelatin. Coomassie brilliant blue (R‐250) was used to stain gels for 45 minutes, and a destaining solution (40% methanol, 10% acetic acid) was applied to highlight the degradation of gelatin by MMPs. Gelatinolytic activity was quantified by measuring the pixel density of individual bands via the FluorChem (FC3) program.

### 2.9 Statistical analysis

Statistical analysis was conducted using SPSS 24.0 statistical software (IBM Corp., Armonk, NY, USA). Differences in the mean of samples were analysed using one‐way ANOVA or Student's *t *test. Statistical data are presented as the mean ± SD (n = 3), and *P* < 0.05 was considered significant.

## 3 RESULTS

### 3.1 Effects of insulin on proliferation, migration and invasion in vitro

**Figure 1:**
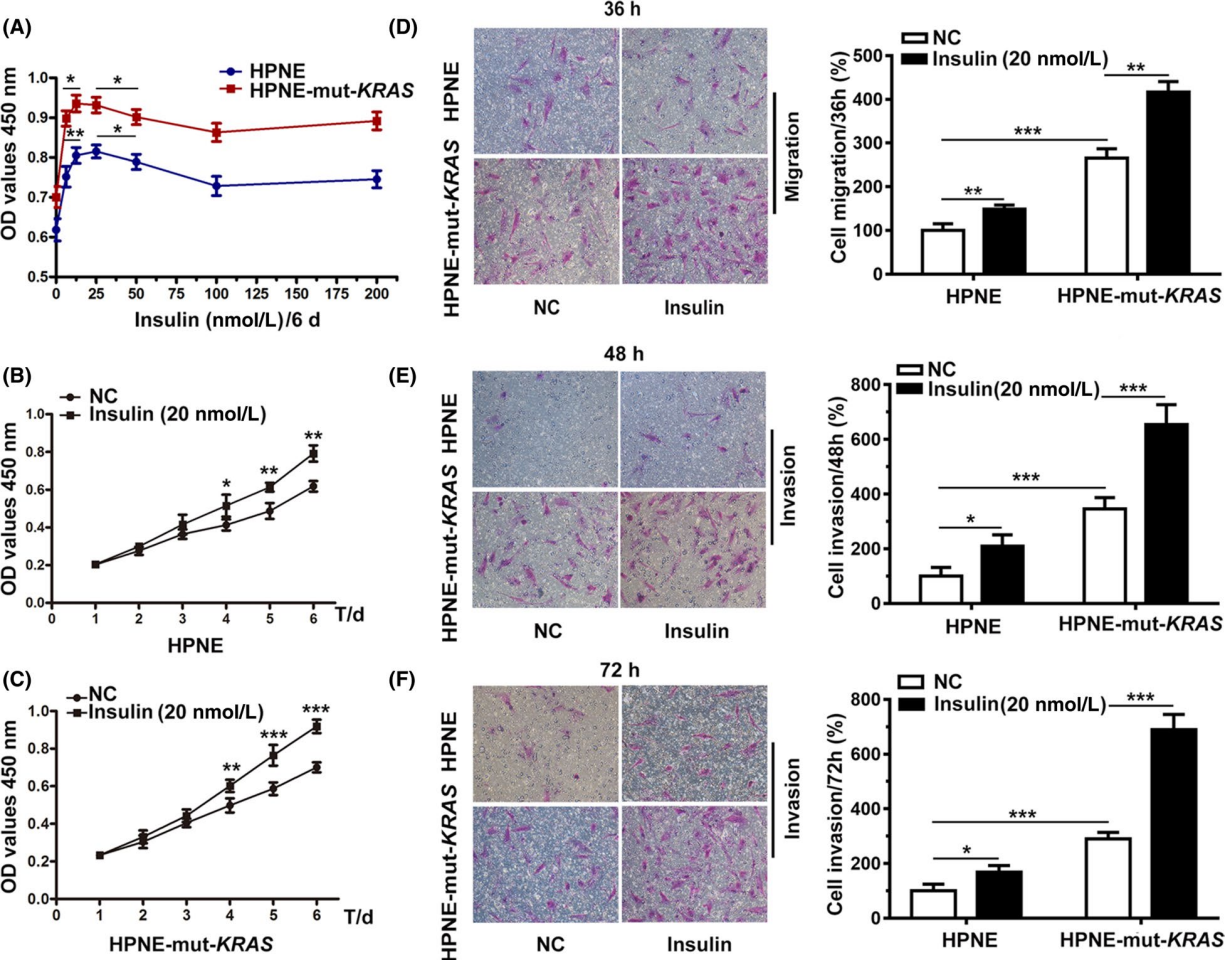
The proliferative and pro‐invasion effects of insulin on HPNE and HPNE‐mut‐*KRAS* over time. A, Cells were treated with or without 6.25, 12.5, 25, 50, 100 or 200 nmol/L of insulin, and cell viability was determined by the CCK‐8 assay. B and C, The CCK‐8 assay showed that insulin promoted HPNE cell proliferation. HPNE cells were seeded in uncoated chambers for the migration assay (D; 36 h) and in Matrigel‐coated chambers for the invasion assay (E and F; 48 and 72 h) in the presence or absence of insulin (20 nmol/L). D‐F, Cells that migrated to the lower compartment and adhering to the bottom surface of the membrane were stained and quantified. The number of migrated HPNE cells without insulin treatment on the bottom surface of the membrane was used as a basal control. The data are presented as the mean ± SD (n = 3). *P < 0.05, **P < 0.01, ***P < 0.001 vs untreated control

A previous study demonstrated that insulin could promote proliferation in immortalized pancreatic ductal cell lines,^19^ so a series of insulin concentrations (0, 6.25, 12.5, 25, 50, 100, 200 nmol/L) were tested in this study to determine the most effective concentration. Concentrations between 12.5 and 25 nmol/L had the greatest impact on cell viability (Figure 1A); therefore, a concentration of 20 nmol/L was used in subsequent experiments. A cell‐counting assay was conducted to exclude the potential impact of cell viability on the pro‐migration and pro‐invasion effects of insulin, and the results showed that insulin had no significant effect on cell viability within 72 hours (Figure 1B,C). The transwell system was then used to evaluate the influence of insulin on the migration and invasion capability of HPNE cells, as well as its oncogenic *KRAS^G12D^* variant. There was increased migration and invasion of HPNE*‐*mut‐*KRAS* cells in comparison with HPNE cells, which could be further enhanced by the additional administration of 20 nmol/L of insulin (Figure 1D‐F).

### 3.2 Involvement of MMP‐2 in insulin‐induced migration and invasion

**Figure 2:**
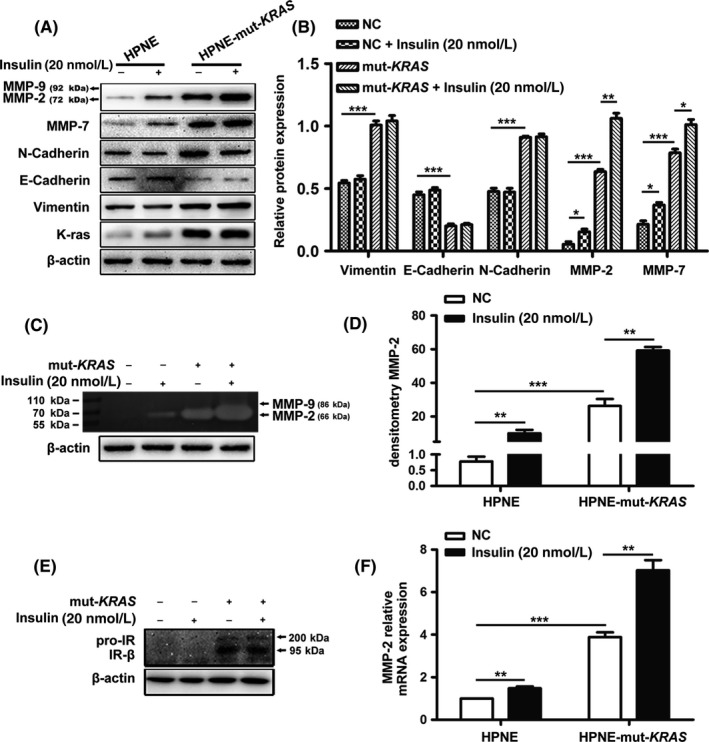
Effects of insulin (20 nmol/L; 24 h) on MMP‐2 expression and gelatinolytic activity in both HPNE cell lines. Western blot and protein expression level of MMP‐2, MMP‐7, E‐cadherin, N‐cadherin, vimentin (A and B) and IR‐β (E) treated with or without insulin (20 nmol/L; 24 h). Protein expression was normalized using β‐actin as a loading control. (F) The mRNA expression of MMP‐2 was investigated using real‐time qRT‐PCR. (C and D) The enzyme activity of MMP‐2 in the culture supernatants was analysed using gelatin zymography. The data are expressed as the mean ± SD (n = 3). *P < 0.05, **P < 0.01, ***P < 0.001 vs untreated control

Both cell lines were incubated with insulin (20 nmol/L) for 24 hours and Western blotting and RT‐qPCR were conducted to determine whether insulin modulates the expression of MMPs and critical molecules in the epithelial‐mesenchymal transition (EMT) process. As shown in Figure 2A,B, the expression of MMP‐2, MMP‐7, vimentin and N‐cadherin was increased in *KRAS* mutated cells, while E‐cadherin was downregulated. Importantly, insulin could significantly increase MMP‐2 expression at both the mRNA and the protein level in *KRAS* mutant cells (Figure 2A,F). The expression of MMP‐7 was slightly elevated after insulin treatment, but no MMP‐9 expression was detected in either cell line (Figure 2A,C). The analysis of the gelatinolytic activity of MMP‐2 (Figure 2C) revealed a remarkable increase in insulin‐induced enzymatic activity of MMP‐2 (active forms; 66 kDa) in both cell lines (Figure 2E). In addition, the expression of the insulin receptor‐beta (IR‐β) was detected in both cell lines. As shown in Figure 2E, *KRAS* mutation induced the increased expression, but insulin stimulation had no effect.

**Figure 3:**
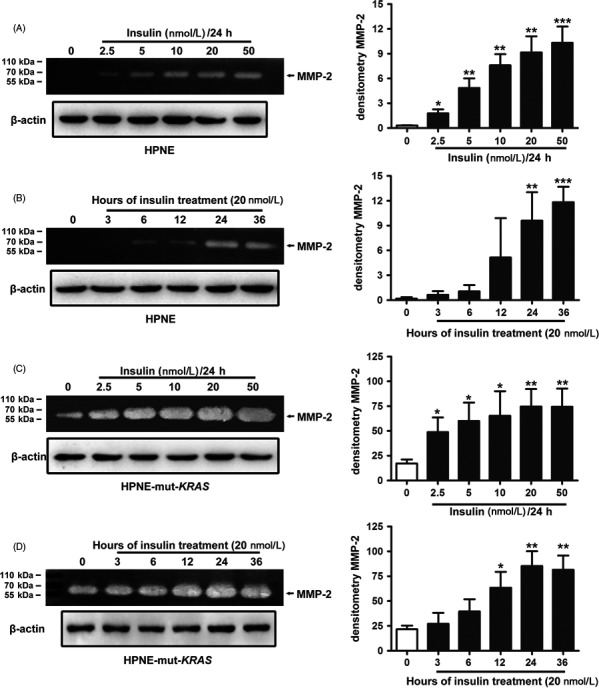
Promotion of MMP‐2 activity by insulin was concentration‐dependent (A and C; 24 h). Activated MMP‐2 increased time‐dependently and was significantly induced following 12 h of stimulation with 20 nmol/L of insulin (B and D). The data are expressed as the mean ± SD (n = 3). *P < 0.05, **P < 0.01, ***P < 0.001 vs untreated control

Further analysis demonstrated that the insulin‐induced MMP‐2 gelatinolytic activity is concentration‐dependent in both HPNE and HPNE*‐*mut‐*KRAS* (Figure 3A,C). The time course study revealed that gelatinolytic activity significantly increased following 12 hours of stimulation with 20 nmol/L of insulin (Figure 3B,D).

**Figure 4:**
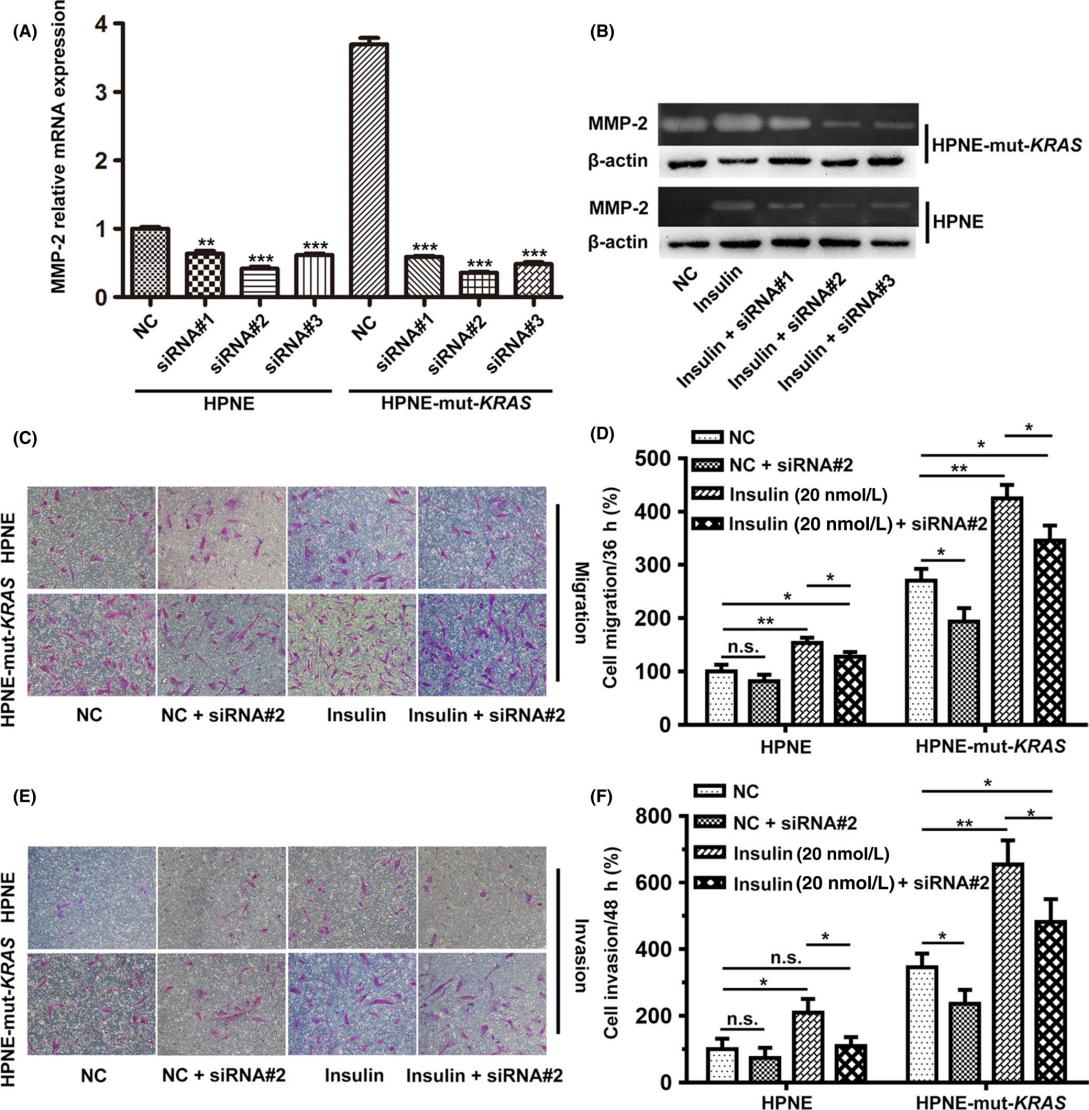
Insulin promoted the migration and invasion activity through the upregulation of MMP‐2 gelatinolytic activity. (A and B) The different interference efficiency of three siRNAs for MMP‐2 was evaluated by qRT‐PCR and gelatin zymography, with both migration (C and D; 36 h) and invasion (E and F; 48 h) capability of the two cell lines suppressed by siRNA#2. Cells that migrated to the lower compartment and adhering to the bottom surface of the membrane were stained and quantified. The number of migrated HPNE cells without insulin treatment on the bottom surface of the membrane was used as a basal control. The data are expressed as the mean ± SD (n = 3). *P < 0.05, **P < 0.01, ***P < 0.001 vs untreated control

It has been suggested that MMP‐2 is involved in the regulation of cell migration and invasion.^20^, ^21^ To further investigate the role of MMP‐2 in the stimulatory effects of insulin on cellular migration and invasion, a blockade study using MMP‐2 siRNA was carried out with insulin treatment. The interference efficiency of three siRNAs was evaluated via qRT‐PCR and gelatin zymography, and siRNA#2 was selected for the following studies (Figure 4A,B). Suppression of MMP‐2 with siRNA can partially abolish the pro‐migration activity through 20 nmol/L of insulin in both HPNE and HPNE*‐*mut‐*KRAS*, and the basic migration activity also decreased after the administration of MMP‐2 siRNA (Figure 4C,D). Similar action of MMP‐2 siRNA in respect of the invasion ability of both cell lines with or without 20 nmol/L of insulin was also observed (Figure 4E,F).

### 3.3 Involvement of insulin receptor in insulin‐induced migration and invasion

**Figure 5:**
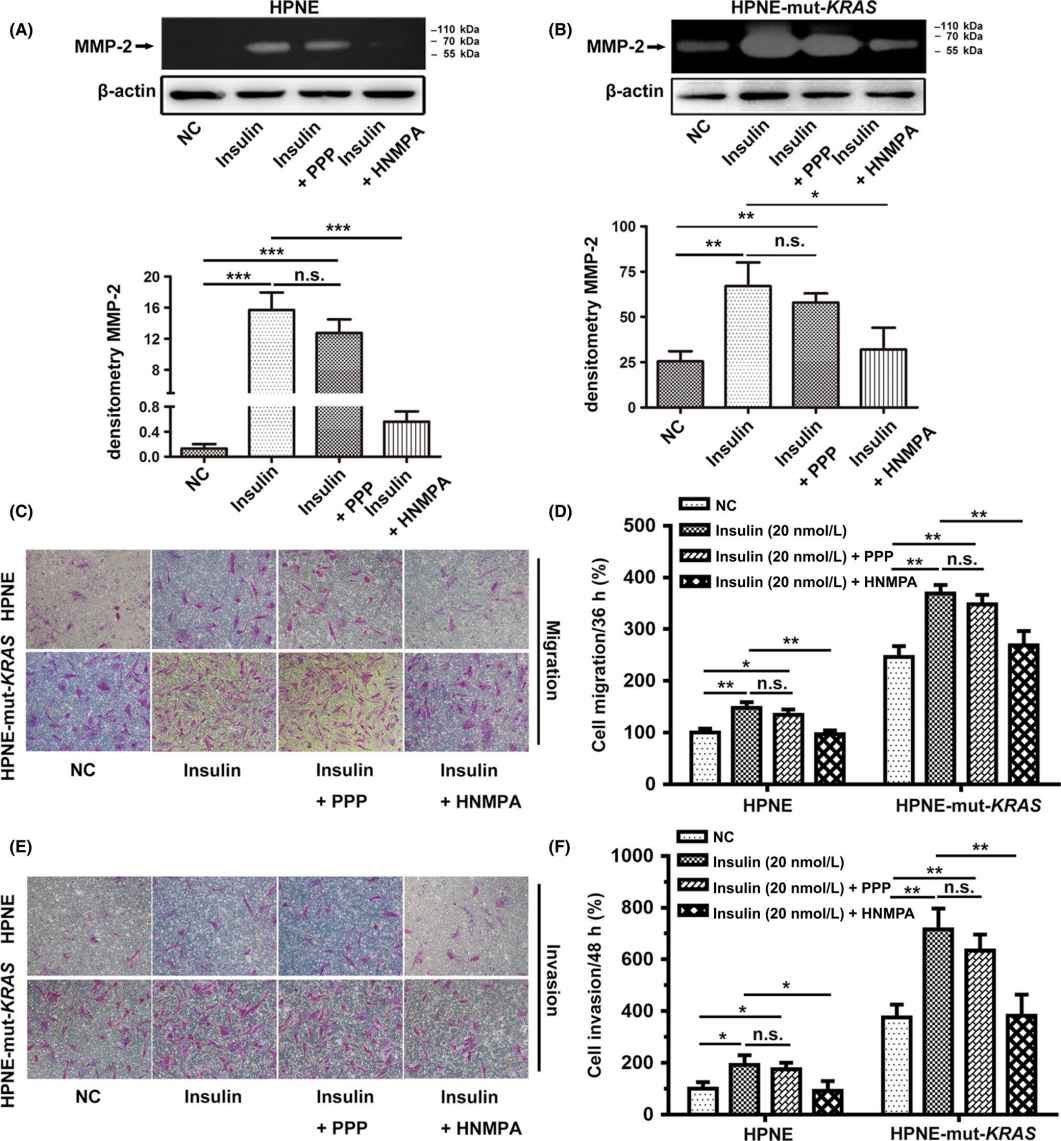
Insulin promoted the migration and invasion of the cells via the IR Representative results of MMP‐2 activity after insulin stimulation with different receptor inhibitors (A and B). Both cell lines were pre‐treated with either HNMPA‐(AM)_3_ (50 μmol/L) or PPP (20 μmol/L), which are specific insulin receptor tyrosine kinase inhibitors and IGF1R inhibitors, respectively, for 4 h (A and B). In transwell experiments, cells were treated with insulin (20 nmol/L) combined with the inhibition of IR or IGF1R, and then the migration assay (C and D; 36 h) and invasion assay (E and F; 48 h) were conducted. Cells that migrated to the lower compartment and adhering to the bottom surface of the membrane were stained and quantified. The number of migrated HPNE cells without insulin treatment on the bottom surface of the membrane was used as a basal control. The data are expressed as the mean ± SD (n = 3). *P < 0.05, **P < 0.01, ***P < 0.001 vs untreated control

To explore the potential mechanism of insulin in promoting MMP‐2 expression, we evaluated whether the insulin receptor or insulin‐like growth factor 1 receptor (IGF1R) was involved in this process. As shown in Figure 5A,B, MMP‐2 activity was completely inhibited by HNMPA‐(AM)_3_ in both cell lines, but not with IGF1R antagonist PPP. Furthermore, transwell experiments were carried out to detect the effects of these two inhibitors on cell migration and invasion (Figure 5C,E). As illustrated in Figure 5D,F, HNMPA‐(AM)_3_, not PPP, completely blocked insulin‐induced migration and invasion in both cell lines. These results showed that insulin activated insulin receptors, which, in turn, increased the expression of MMP‐2, as well as promoting the migration and invasion of the cells.

### 3.4 Involvement of PI3K/AKT and ERK1/2 MAPK pathways in insulin‐induced migration and invasion

**Figure 6:**
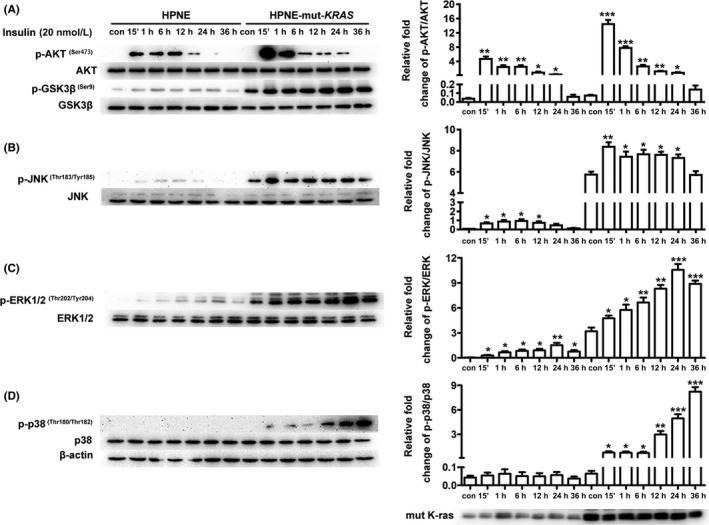
Effect of insulin on the activity of PI3K/AKT and MAPK signalling in HPNE and HPNE‐mut‐*KRAS* cells. Cells were exposed to 20 nmol/L of insulin for various amounts of time. Whole‐cell lysates were extracted for the detection of the protein levels of (A) p‐AKT (Ser473) and AKT, p‐GSK3β (Ser9) and GSK3β, (B) p‐JNK (Thr183/Tyr185) and JNK, (C) p‐ERK1/2 (Thr202/Tyr204) and ERK1/2, and (D) p‐p38 (Thr180/Tyr182) and p38 by Western blotting. β‐Actin served as the loading control. The data are expressed as the mean ± SD (n = 3). *P < 0.05, **P < 0.01, ***P < 0.001 vs untreated control

It has been suggested that insulin can activate classical PI3K/AKT and MAPK pathways.^22^, ^23^ In this study, we found that the phosphorylation levels of GSK3β and MAPK pathways were upregulated in the *KRAS* mutant cells and the activating effect of insulin on PI3K/AKT/GSK3β, MEK/ERK signalling pathway had been significantly augmented by the introduction of mutant *KRAS* gene (Figure 6A). In addition, there was also upregulated phosphorylation of JNK, ERK1/2 and p38 in both cells with insulin stimulation (Figure 6B‐D). Taken together, these results demonstrated that insulin can activate the PI3K/AKT and MAPK pathways in both cell lines.

**Figure 7:**
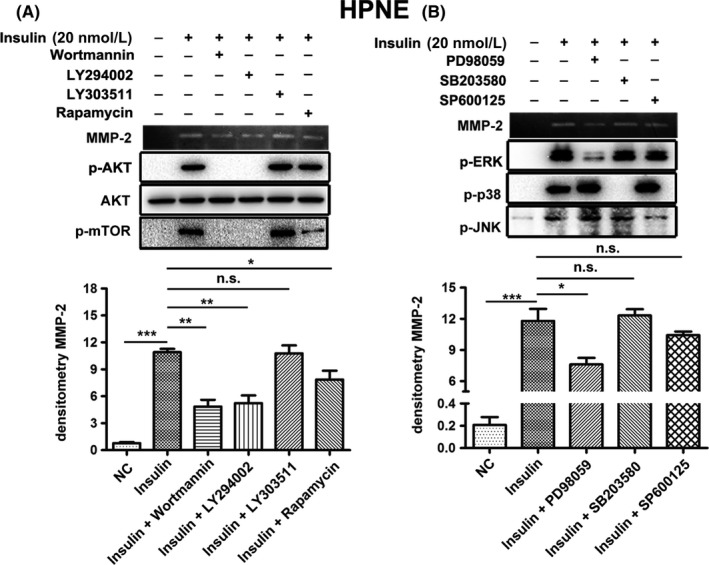
A, Inhibition of the PI3K/AKT pathway with wortmannin (50 nmol/L) or LY294002 (20 μmol/L) inhibited insulin‐mediated (20 nmol/L; 24 h) MMP‐2 activation in HPNE cells. LY303511 (20 μmol/L) was used as a negative control. Rapamycin (25 ng/mL), an inhibitor of mTOR/p70^s6^ kinase signalling, also affected insulin‐induced MMP‐2 activation. B, The MEK1 inhibitor PD98059 (50 μmol/L) significantly inhibited insulin‐induced (20 nmol/L; 24 h) MMP‐2 gelatinolytic activity, whereas the JNK inhibitor SP600125 (15 μmol/L) and the p38 inhibitor SB20350 (25 μmol/L) had no effect. Cells were pre‐incubated with inhibitors for 4 h before insulin treatment. The data are expressed as the mean ± SD (n = 3). *P < 0.05, **P < 0.01, ***P < 0.001 vs untreated control

**Figure 8:**
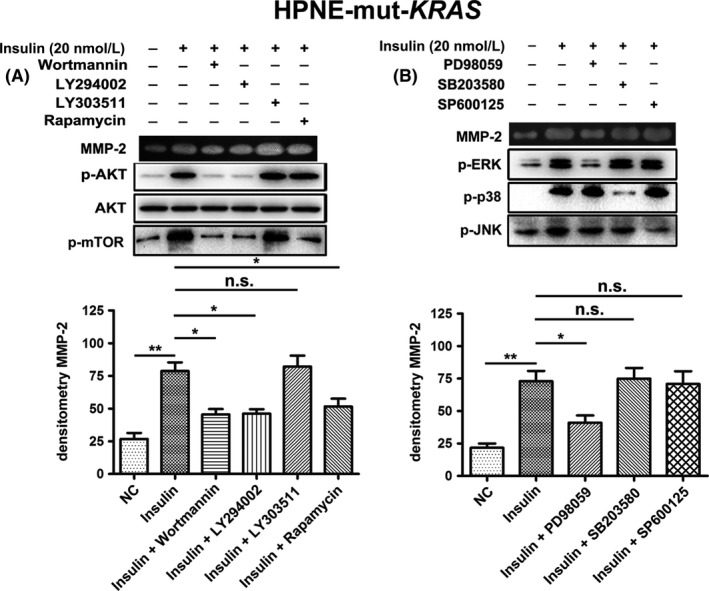
A, Inhibition of the PI3K/AKT pathway with wortmannin (50 nmol/L) or LY294002 (20 μmol/L) inhibited insulin‐mediated (20 nmol/L; 24 h) MMP‐2 activation in HPNE‐mut‐*KRAS* cells. LY303511 (20 μmol/L) was used as a negative control. Rapamycin (25 ng/mL), an inhibitor of mTOR/p70^s6^ kinase signalling, also affected insulin‐induced MMP‐2 activation. B, The MEK1 inhibitor PD98059 (50 μmol/L) significantly inhibited insulin‐induced (20 nmol/L; 24 h) MMP‐2 gelatinolytic activity, whereas the JNK inhibitor SP600125 (15 μmol/L) and the p38 inhibitor SB20350 (25 μmol/L) had no effect. Cells were pre‐incubated with inhibitors for 4 h before insulin treatment. The data are expressed as the mean ± SD (n = 3). *P < 0.05, **P < 0.01, ***P < 0.001 vs untreated control

To examine whether PI3K/AKT and MAPK pathways were associated with the insulin‐induced increase in MMP‐2 gelatinolytic activity, HPNE cells were pre‐treated with PI3K/AKT and MAPK (ERK, JNK and p38)‐specific inhibitors before exposure to insulin. Compared with insulin treatment alone, wortmannin or LY294002 (a PI3K‐specific inhibitor), rapamycin (a mTOR‐specific inhibitor) and PD98059 (a MEK1‐specific inhibitor) significantly inhibited the insulin‐dependent increase in MMP‐2 gelatinolytic activity, while LY303511 (a negative control for LY294002), SB203580 (a p38‐specific inhibitor) and SP600125 (a JNK‐specific inhibitor) had no effect (Figure 7A,B). Similar results were observed in the HPNE*‐*mut‐*KRAS* cells (Figure 8,B). To further investigate whether the PI3K/AKT pathway has a crosstalk with MEK/ERK pathway for the obviously upregulated phosphorylation of PI3K/AKT pathway in the *KRAS* mutant cells, we treated both cells with a MEK1 inhibitor (PD98059) and a PI3K inhibitor (LY294002), respectively. We observed a slight induction of AKT pathway in response to MEK1 inhibition in both cells, while inhibition of PI3K had no effect on MEK/ERK pathway (Figure S1).

**Figure 9:**
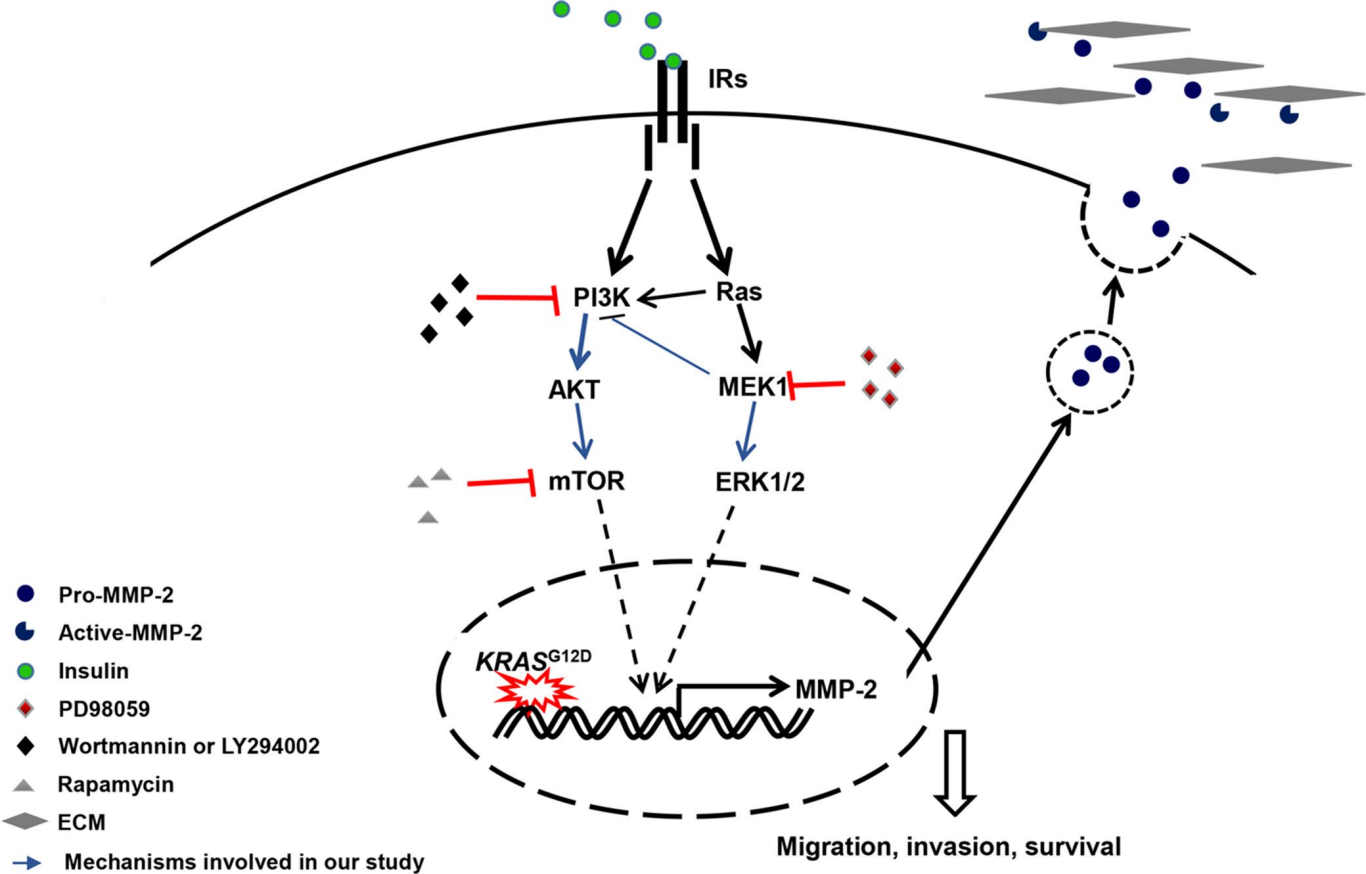
The PI3K/AKT and ERK MAPK pathways are involved in the regulation of MMP‐2. Schematic representation of the proposed mechanism of MMP‐2 expression in both HPNE cell lines. Phosphorylation of the insulin receptors by insulin drives the downstream activation of PI3K/AKT and ERK. Increased ERK or PI3K/AKT activity results in enhanced MMP‐2 gelatinolytic activity involved in migration, invasion and tumour progression

A schematic representation of the proposed mechanism of MMP‐2 expression in both HPNE cell lines was demonstrated that the activation of insulin receptors by insulin drives the downstream activation of PI3K/AKT and ERK. Increased ERK or PI3K/AKT activity results in enhanced MMP‐2 gelatinolytic activity involved in migration, invasion and tumour progression (Figure 9).

## 4 DISCUSSION

Type 2 diabetes is a systematic disease characterized by hyperinsulinemia and hyperglycaemia. Epidemiological evidence suggests that type 2 diabetes can increase the risk of multiple cancers and that patients who have a history of diabetes for more than 5 years have a significantly increased risk of pancreatic cancer. Insulin is secreted by the pancreatic β cells and transported through the portal vein. Moreover, the pancreas is exposed to higher concentrations of endogenous insulin than peripheral blood and previous study has demonstrated that the physiological concentration of insulin between 0.2 to 20 nmol/L can protect pancreatic cells from apoptosis via insulin receptor.^24^, ^25^ Therefore, the insulin concentration used in our study is physiologically attainable in pancreas. Numerous studies have suggested that insulin, rather than blood glucose, is an independent risk factor for pancreatic cancer.^16^, ^17^ Indeed, insulin can promote pancreatic cancer cell viability and cancer progression.^19^, ^26^ Therefore, this study explored the role of physiological concentration of insulin in the malignant progression of human pancreatic duct‐derived cells, as well as clarifying the underlying mechanism in vitro.

The extracellular matrix (ECM) plays an important role in maintaining the integrity of tissue structure, and its degradation and basement membrane breakdown are essential for the early stage of local invasive events. There is considerable evidence that MMPs, particularly MMP‐2, play a vital role in promoting tumour invasion, enabling the disintegration of epithelial tissue and cell migration or invasion.^21^, ^27^, ^28^ Moreover, the decomposition of ECM leads to the release of ECM‐bound factors, which, in turn, are involved in the regulation of pathological parameters,^29^ angiogenesis or lymphangiogenesis,^30^, ^31^ chronic inflammation,^32^ metastasis and tumour growth.^33^, ^34^ Importantly, the active MMP isozyme is highly expressed in PDAC cells^35^, ^36^ and serum levels of MMP‐2 have prognostic significance in pancreatic cancer patients.^37^ MMP‐2 expression was associated with microvessel density in pancreatic cancer, along with higher lymph node metastasis.^38^ Taken together, these findings suggest that MMP‐2 may act as a key regulator in the progress of pancreatic tumorigenesis. Furthermore, the most recent research has demonstrated that circulating MMP‐2 levels in diabetics were significantly increased.^39^

The *KRAS* mutation is a critical determinant in the early stage of pancreatic ductal adenocarcinoma and is able to drive mature pancreatic cells to de‐differentiate into duct‐like cells and, ultimately, PDAC.^4^ Numerous studies have focused on the role of *KRAS* mutation in promoting tumorigenesis. In vitro study, microinjection of mutant K‐Ras^G12V^ into primary pancreatic ductal cells can induce a phenotypic conversion and an increase in proliferation.^40^ Oncogenic *KRAS^G12D^* can continuously activate its downstream pathways, which lead to a series of neoplastic related events, including promotion of proliferation, suppression of apoptosis, changing metabolic pathways, remodelling the microenvironment, evasion of the immune response and cell migration and metastasis.^41^ Importantly, a previous study in conditional *KRAS^G12D^* mouse model feeding with high‐fat high‐calorie diet has demonstrated that metabolic syndrome, with hyperinsulinemia as one of its characteristics, could accelerates the development of mPanINs in *KRAS^LSL‐G12D^*‐pdx1‐Cre mice.^15^ In addition, the relationship between insulin and *KRAS* mutation has also been studied in lung cancer and it is reported that insulin/IGF1 signalling is important for lung cancer initiation after *KRAS* mutation.^42^ Therefore, *KRAS* mutation is essential for the occurrence of PDAC via increasing architecture and cytological atypia. In this study, we attempted to model the stages of PDAC in vitro using HPNE to represent the “normal” *KRAS* wild‐type baseline stage and HPNE*‐*mut‐*KRAS* to represent a *KRAS* mutant stage.^43^, ^44^ In this model, insulin was shown to be a potent inducer of MMP‐2 gelatinolytic activity, enhancing the invasion and migration of both human pancreatic cell lines, and MMP‐2 siRNA significantly reversed the insulin‐induced migration and invasion. It is of note that MMP‐2 expression in *KRAS* mutation cell lines was much higher than that in normal cells,^45^ and this upregulation was much more pronounced after insulin treatment, suggesting the synergistic effects of *KRAS* mutation and insulin in promoting cellular invasion. In addition to the abovementioned roles of MMP‐2 in promoting tumorigenesis, the physiological level of MMP‐2 plays an important role in preventing the accumulation and maintaining the dynamic balance of ECM. And in vivo study proved that mice with the *KRAS* mutation could develop mPanIN lesions.^15^ We hypothesized that MMP‐2 is more likely to participate in the dynamic regulation of ECM remodelling and chronic inflammation in diabetic patients without *KRAS* mutation. Conversely, elevated MMP‐2 probably leads to a higher level of PanIN lesions in mutant patients. Interestingly, we found that siRNA#1 can effectively reduce MMP‐2 mRNA but partly reduced insulin‐induced MMP‐2 gelatinolytic activity. We reviewed the literature and found that different transcripts have different efficiencies when translated into proteins. The efficiency of this process is influenced by many factors, including the efficiency of post‐transcriptional translation, protein modification and degradation, as well as environmental factors.^46^, ^47^ In our experiments, we detected MMP‐2 gelatinolytic activity in the conditioned medium, which was also affected by the exocrine function of HPNE cell lines. Therefore, MMP‐2 gelatinolytic activity in conditioned medium can be affected by multiple factors in our study.

The mechanism for the regulation of MMP‐2 gelatinolytic activity in this study remains largely unknown. Downregulation of the insulin receptor can inhibit cancer cell proliferation and metastasis, altering downstream signalling in vivo.^48^ It has been shown that insulin receptors have a high affinity for insulin (±10^−10^ mol/L), while IGF1R has a higher affinity for IGF1 and IGF2 (±10^−10^ mol/L), which is 100‐fold higher than that for insulin.^49^ Our data suggest that the *KRAS* mutation, rather than insulin, can induce an increased expression of insulin receptors, the mechanism of which warrants further study. The use of an insulin receptor tyrosine kinase inhibitor significantly inhibited migration, invasion induced by insulin and MMP‐2 gelatinolytic activity. However, inhibition of IGF1R did not have a significant effect. These results suggest that insulin can upregulate MMP‐2 via its classical receptor, independent of IGF1R in human pancreatic cells. As mentioned above, MMP‐2 plays an important role in the development of PDAC. Moreover, several studies have also shown that overexpression of MMP‐2 is associated with the progression of multiple cancers, as well as metastases.^50^, ^51^ Inhibiting the expression of MMP‐2 can significantly suppress tumour progression.^52^ Therefore, the IR tyrosine kinase may serve as a promising therapeutic target for preventing pancreatic carcinogenesis. Linsitinib (OSI‐906), a dual inhibitor of insulin receptor and IGF1R, for solid tumours has been examined in clinical trials.^53^ It may be possible to prevent pancreatic cancer by targeting high‐risk groups (eg with a family history) in patients with long‐term type 2 diabetes in the future. However, it is of note that a high concentration of insulin can lead to changes in the EMT phenotype of breast cancer cells via IGF1R.^54^ In addition, we found that these cell models derived from exocrine tissue required relatively higher doses of insulin to elicit response via IGF1R when compared to physiological insulin dose.^19^ This finding suggests the possibility that the pancreatic precursor cancer cells may react to physiological concentrations insulin via insulin receptor, and high levels of insulin would be expected to activate IGF1R. Nonetheless, at the physiological concentration of 20 nmol/L used in this study, there was no significant change in the expression of EMT‐related molecules, but this requires further investigation.

Increasing evidence has suggested that insulin can activate classical PI3K/AKT and MAPK signalling via binding to insulin receptors.^19^, ^24^, ^55^ Additionally, it has been shown that MMP‐2 expression is critically mediated by the MAPK or PI3K/AKT pathways in various cell types.^56^, ^57^ Our experimental results showed that the phosphorylation of PI3K/AKT and ERK1/2 MAPK signalling molecules in normal HPNE cells is time‐dependent and that phosphorylated levels are higher in the *KRAS* mutant cells. In addition, insulin also increased the phosphorylation of JNK and p38 MAPKs. Further study revealed that the PI3K/AKT pathway has a crosstalk with MEK/ERK pathway (Figure S1). These results suggested a feedback also observed in other pancreatic cell lines.^63^ Our results suggested that the expression of insulin receptors was upregulated in the *KRAS* mutant cells which may be responsible for the notably phosphorylation of AKT. And, previous studies have also observed an overexpression of IRS1 and IRS2 which could transmit the activation of IR during pancreatic carcinogenesis.^64^, ^65^ Besides, Chalabi‐Dchar et al^66^ found that *KRAS* mutation could decrease the expression of somatostatin receptor subtype 2, thereby weakening its inhibition of the PI3K/AKT pathway in the *KRAS* mutant HPNE cells. Importantly, pre‐incubation with a MEK1 inhibitor (PD98059) after insulin treatment partly downregulated MMP‐2 gelatinolytic activity. Therefore, the ERK1/2 MAPK pathway response to cell invasion and migration requires insulin stimulation in human pancreatic cells. Furthermore, PI3K/AKT signalling and its downstream signals are crucial in the development of cancer, participating in the regulation of carcinogenesis, angiogenesis, cell cycle progression and apoptosis.^67^, ^68^ mTOR is a significant downstream protein of PI3K/AKT pathway, and activation of mTOR by p‐AKT can upregulate the expression of MMP‐2 in multiple tumours, including pancreatic cancer.^70^, ^71^ The present study provided information that the insulin‐promoted MMP‐2 gelatinolytic activity was upregulated partly through PI3K/AKT/mTOR signalling. Taken together, our findings suggest that insulin‐induced activation of ERK1/2 MAPK and PI3K/AKT/mTOR signalling may be involved in the invasion and migration through the upregulation of MMP‐2 gelatinolytic activity. However, the mechanism by which insulin interacts with these two signalling pathways causing cell invasion and migration regulated by MMP‐2 is unclear and requires further in vivo investigation.

In conclusion, this study demonstrated that insulin regulated MMP‐2 gelatinolytic activity via its “metabolic” PI3K/AKT and “mitogenic” ERK1/2 signalling pathways in immortalized human pancreatic ductal cell lines, as well as the synergistic effect of hyperinsulinemia and *KRAS* mutation in the early stage of pancreatic cancer. Consequently, the induction of MMP‐2 by insulin may contribute to the degradation of ECM, the breakdown of the basement membrane, increased local infiltration and distant metastasis, which may explain the increased incidence of pancreatic cancer in patients with hyperinsulinemia and type 2 diabetes.
