# Supplementary figures and images for: Insulin promotes invasion and migration of KRASG12D mutant HPNE cells by upregulating MMP‐2 gelatinolytic activity via ERK‐ and PI3K‐dependent signalling
Source: Cell Prolif. 2019 Mar 5;52(3):e12575. doi: 10.1111/cpr.12575 (PMC6536446; doi:10.1111/cpr.12575)

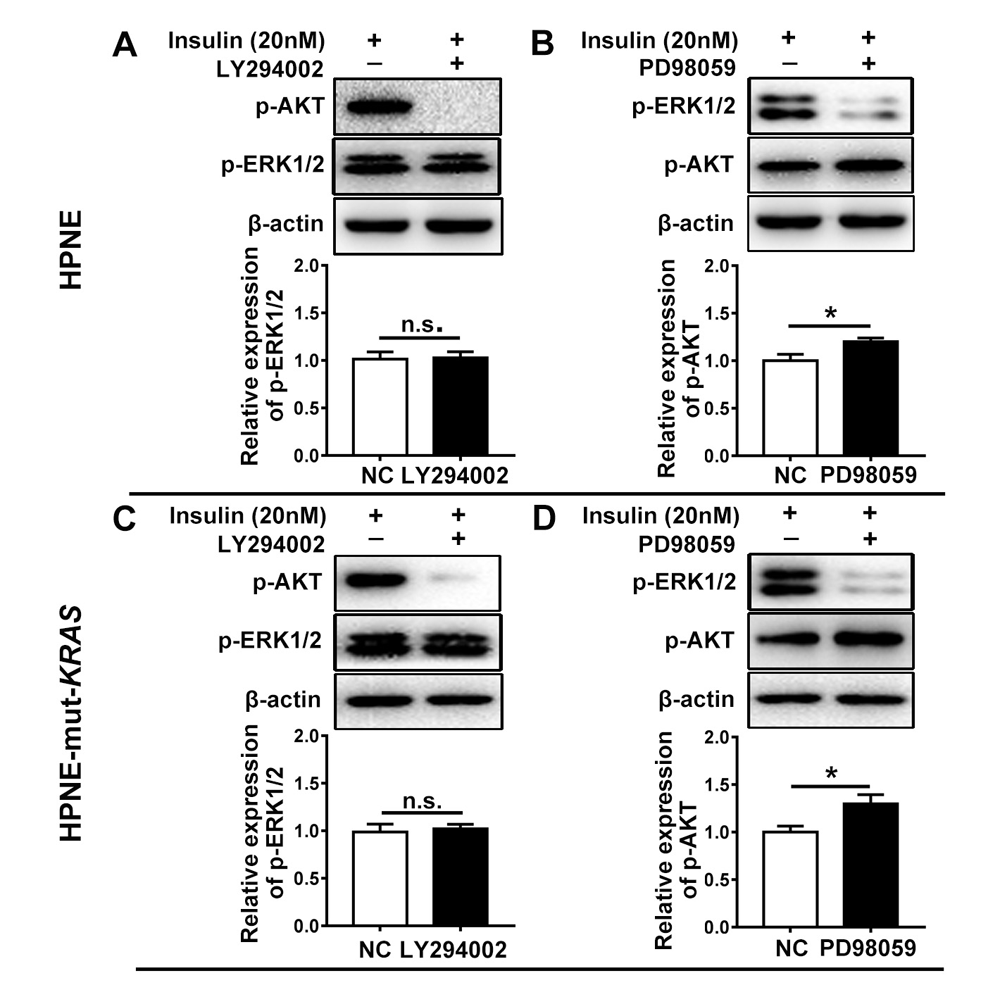

Supplement: Supplementary file 1 [file CPR-52-e12575-s001.tif]
